# *In Vivo* Imaging of Transiently Transgenized Mice with a Bovine Interleukin 8 (CXCL8) Promoter/Luciferase Reporter Construct

**DOI:** 10.1371/journal.pone.0039716

**Published:** 2012-06-28

**Authors:** Fabio Franco Stellari, Valentina Franceschi, Antonio Capocefalo, Marcello Ronchei, Fabrizio Facchinetti, Gino Villetti, Gaetano Donofrio

**Affiliations:** 1 Dipartimento di Salute Animale, Sezione di Malattie Infettive degli Animali, Università di Parma, Parma, Italy; 2 Chiesi Farmaceutici S.p.A, Parma, Italy; Auburn University, United States of America

## Abstract

One of the most remarkable properties of interleukin 8 (CXCL8/IL-8), a chemokine with known additional functions also in angiogenesis and tissue remodeling, is the variation of its expression levels. In healthy tissues, IL-8 is barely detectable, but it is rapidly induced by several folds in response to proinflammatory cytokines, bacterial or viral products, and cellular stress. Although mouse cells do not bear a clear homologous IL-8 gene, the murine transcriptional apparatus may well be capable of activating or repressing a heterologous IL-8 gene promoter driving a reporter gene. In order to induce a transient transgenic expression, mice were systemically injected with a bovine IL-8 promoter–luciferase construct. Subsequently mice were monitored for luciferase expression in the lung by *in vivo* bioluminescent image analysis over an extended period of time (up to 60 days). We demonstrate that the bovine IL-8 promoter–luciferase construct is transiently and robustly activated 3–5 hours after LPS and TNF-α instillation into the lung, peaking at 35 days after construct delivery. Bovine IL-8 promoter–luciferase activation correlates with white blood cell and neutrophil infiltration into the lung. This study demonstrates that a small experimental rodent model can be utilized for non-invasively monitoring, through a reporter gene system, the activation of an IL-8 promoter region derived from a larger size animal (bovine). This proof of principle study has the potential to be utilized also for studying primate IL-8 promoter regions.

## Introduction

Severe respiratory diseases in ruminants and human are characterized by pneumonia with extensive infiltration of polymorphonuclear inflammatory cells (PMNs) in small airways and alveoli, by the accumulation of fibrinous oedema in the alveoli, pleura surface and interlobular septa, by hemorrhage, vascular thrombosis and coagulative parenchymal necrosis of the lung [1], [2].

Several bacteria and viruses overcoming host defense mechanisms and proliferating in the lung are the main triggering factors of lung inflammation and polymorphonuclear leukocytes (PMNs) are directly and indirectly responsible for most of the lung pathologies and lesions [2], [3]. Mobilization of PMNs in the inflamed lung is modulated by the interactions of leucocytes and cytokines. During the acute-phase response of the pneumonic process caused by infectious agents in ruminants and human, the inflammatory cytokines such as IL-1β, TNF-α and IL-8, secreted by a variety of immune and non immune cell types, induce a strong PMNs mobilization and an increased inflammatory response [2], [3]. PMNs mobilization does not always effectively combat the infection and may also contribute to the development of lesions in the lung. During the development of lung inflammation IL-1β and TNF-α are initially produced, thereby inducing a strong IL-8 secretion, the most potent PMNs chemotactic and activating factor. IL-1β, TNF-α and especially IL-8, promote PMNs-mediated tissue injury by stimulating degranulation of PMNs and extracellular release of arachidonic acid metabolites, toxic oxygen radicals and proteolytic enzymes [4]. Hence, IL-8 could be considered a downstream event that is dependent, at least in part, on the prior secretion of early response cytokines such as IL-1β and TNF-α [1], [2], [4] and this assumption has very important implications for therapeutic strategies directed toward the modulation of inflammatory cytokines. First of all pharmacologic molecules inhibiting or antagonizing the biological effect of inflammatory cytokines should be mainly directed toward IL-8. Next, anti-cytokine drugs should be administrated very early to prevent irreversible lung lesions [5], [6].

Interleukin-8 (CXCL8/IL-8) is secreted by a variety of immune and non immune cell types. The human chemokine IL-8 is an 8.5-kDa C-X-C chemokine member of the CXC chemokine family, which acts through the G protein-coupled receptors CXCR1 and CXCR2. IL-8/CXCL8 complex is capable of activating the expression of adhesion molecules in endothelial cells [7] and is an important chemoattractant for neutrophils [8] as well as T-cells [9] and monocytes [10]. In neutrophils, in particular, IL-8 induces shape changes, exocytosis of stored proteins, and the respiratory burst, resulting in the release of superoxide anions and hydrogen peroxide. IL-8 is also involved in a wide variety of pathological processes, including host defenses against bacterial infections, angiogenesis and immune disorders [11]. One of the most remarkable property of IL-8 is the rapid variation of its expression levels in response to numerous stimuli. In healthy tissues, IL-8 is barely detectable, but it is rapidly induced in response to pro-inflammatory cytokines such as tumor necrosis factor alpha (TNF-α) or IL-1, bacterial or viral products, and cellular stress.

Different signaling pathways coordinately regulate IL-8 transcription as well as mRNA stabilization in response to external stimuli. Maximal IL-8 amounts are generated by a combination of three different mechanisms: first, derepression of the gene promoter; second, transcriptional activation of the gene by nuclear factor-kappaB (NF-*k*B) and JUN-N-terminal protein kinase (JNK) pathways; and third, stabilization of the mRNA by the p38 mitogen-activated protein kinase pathway. This complex regulation renders cells capable of rapidly increasing and, at the same time, to fine-tuning the amount of IL-8 secreted, thereby tightly controlling the extent of leukocytes attracted to the site of tissue injury.

**Figure 1:**
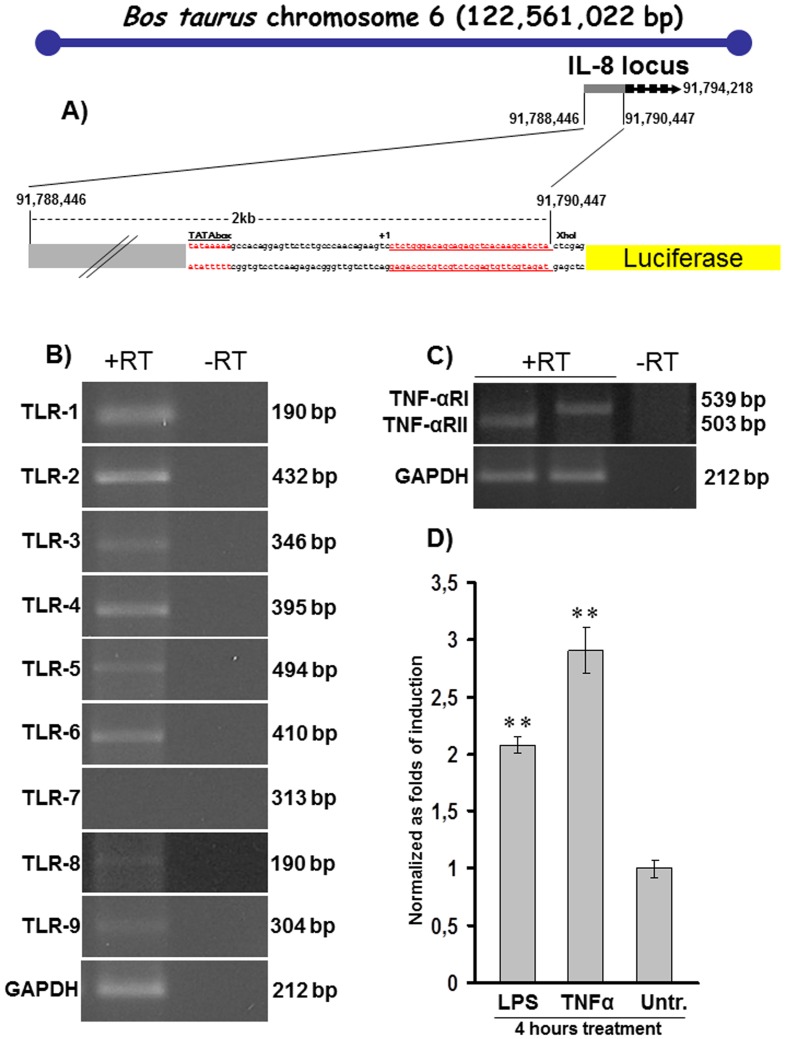
*In vitro* characterization of bIL-8-Luc reporter construct. **A**) Schematic diagram (not to scale) of the *Bos taurus* chromosome 6 with the IL-8 gene locus comprising promoter and exons annotated by numbers. The 2 kb IL-8 promoter sequence, comprising the putative TATA box, the transcriptional start site (+1) and the UTR (red underlined) was cloned into the pGL3 basic vector in front of the open reading frame of the Luciferase reporter. **B**) RT-PCR products of TLRs from 1 to 9 and their corresponding amplicon size. GAPDH was used as an internal control and the amplification performed in the absence of reverse transcriptase (-RT) as a negative control. **C**) RT-PCR products of TNF-αRI,II and their corresponding amplicon size. GAPDH was used as an internal control and the amplification performed in the absence of reverse transcriptase (-RT) as a negative control. D) IL-8 promoter activation after treatment of bIL-8-Luc transfected LA-4 cells with LPS or TNF-α, along with the untreated control (Untr.). Each experiment was done in quadruplicate, and each point represents the mean ± standard deviation of three experiments. Data were expressed as folds of induction (2,1 and 2,8 for LPS and TNF-α respectively) over vehicle-treated cells and statistical differences were tested by One Way ANOVA followed by Dunnet’s post hoc test for group comparisons. Results are reported as mean ± SD and significance attributed when *P*<0.05 (*) or *P*<0.01(**).

Although post-transcriptional regulation of IL-8 expression has been shown to be important, transcriptional regulation linked to the activation of pro-inflammatory molecule receptors, such as TLRs, IL-1 and TNF-α receptors, remains crucial in modulating IL-8 expression [12]. Available data suggested that the NF-*k*B and JNK pathways are indispensable for the transcriptional regulation of IL-8 expression. Using the chromatin immune-precipitation technique, Nissen and Yamamoto demonstrated the binding of p65 NF-*k*B to the IL-8 promoter and the subsequent formation of a TNF-α-stimulated pre-initiation complex containing inducible phosphorilated RNA polymerase II [13], [14]. Therefore, establishing an *in vivo* molecular read-out to monitor IL-8 induction following a specific inflammatory stimulus would be of great value for testing new strategies directed toward the transcriptional modulation of IL-8 production. Although the manifestation of lung diseases in ruminants overlaps in most respect the manifestation of lung diseases in human [2], [3], the use of ruminants (bovine, sheep or goat) as animal models is very costly and demanding in terms of maintenance and the availability of advanced genetic or immunologic resources is significantly limited when compared with those available for rodent studies. On the other hand, no clear-cut IL-8 homolog in rat or mouse has been identified so far, although MIP-2 and KC may be functional homologs of IL-8 in mice [15]. Furthermore, IL-8 binds to two types of IL-8 receptors in humans, while mouse has only one potential IL-8 receptor (homolog of human CXCR2). Although mice lack an exact homolog of IL-8, the murine CXCR2 homolog is activated by other neutrophils chemoattractants, namely MIP-2 and KC. Indeed, gene targeting of CXCR2 in mice exhibited inhibition of neutrophil infiltration into inflamed tissue [16] and mice transiently overexpressing IL-8 exhibit excessive accumulation of neutrophils in the microcirculation of the lung, liver, and spleen [15]. Based on the above information it was hypothesized that, although mouse cells do not bear a clear homologous of IL-8 gene, murine transcriptional apparatus may nonetheless be capable of activating or repressing a heterologous IL-8 gene promoter driving a reporter gene. The possibility of monitoring in small rodents *in vivo* luciferase reporter genes under the control of cytokine promoters derived from other species would be of great value for studying the pathophysiology of inflammatory responses as well as to test interventions aimed at modulating such responses. In the present study this hypothesis was successfully tested by utilizing a molecular imaging approach based on a previously well characterized bovine IL-8 promoter/luciferase reporter construct [17] in mice. This study paves the way to non-invasive imaging approaches for studying mechanisms and signaling pathways underlying regulation of IL-8 expression.

## Results

### The Bovine IL-8 Promoter is Responsive in Mouse Lung Cells *in vitro*

The bovine IL-8 promoter, mapped on *Bos taurus* chromosome 6 (http://www.ncbi.nlm.nih.gov/mapview/maps.cgitaxid=9913&chr=6&query=uid(207248,3567299)&QSTR=280828%5Bgene%5Fid%5D&maps=gene_set&cmd=focus), was previously cloned from bovine genomic DNA template and its activation at the transcriptional level tested with a luciferase reporter construct bIL-8-Luc. The luciferase construct was generated by sub-cloning the 2030 bp IL-8 promoter in front of a pGL3 Luciferase reporter vector [17]. The capability of mouse cells to trans-activate the bovine IL-8 promoter/luciferase reporter construct (bIL-8-Luc, **Fig. 1A**) was first tested in an *in vitro* model. The expression of Toll Like receptors (TLRs,) was evaluated by RT-PCR in the LA-4 cell line, a mouse epithelial cell line from lung, TLR 1, 2, 3, 4, 5, 6, 8 and 9 were detectable, while no expression of TLR7 was identified (**Fig. 1B**). Secondly, TNF-α and TNF-α receptor (TNF-αR) I and II were both found expressed in LA-4 cells (**Fig. 1C**). Subsequently, LA-4 cells were transiently transfected by electroporation with the bIL-8-luc reporter construct and 24 h post transfection the cells were treated with TNF-α or LPS. In bIL-8-luc transfected LA-4 cells, a 4 hour treatment with TNF-α increased luciferase signal up to 3 folds, while, similarly, a 4 hour treatment with LPS elicited a 2-fold induction (**Fig. 1D**). Signal intensity was expressed as folds of induction over the bIL-8-luc transfected vehicle-treated LA-4 cells. Results are reported as mean ± SD and significance attributed when *P*<0.05 (*) or *P*<0.01(**).

**Figure 2:**
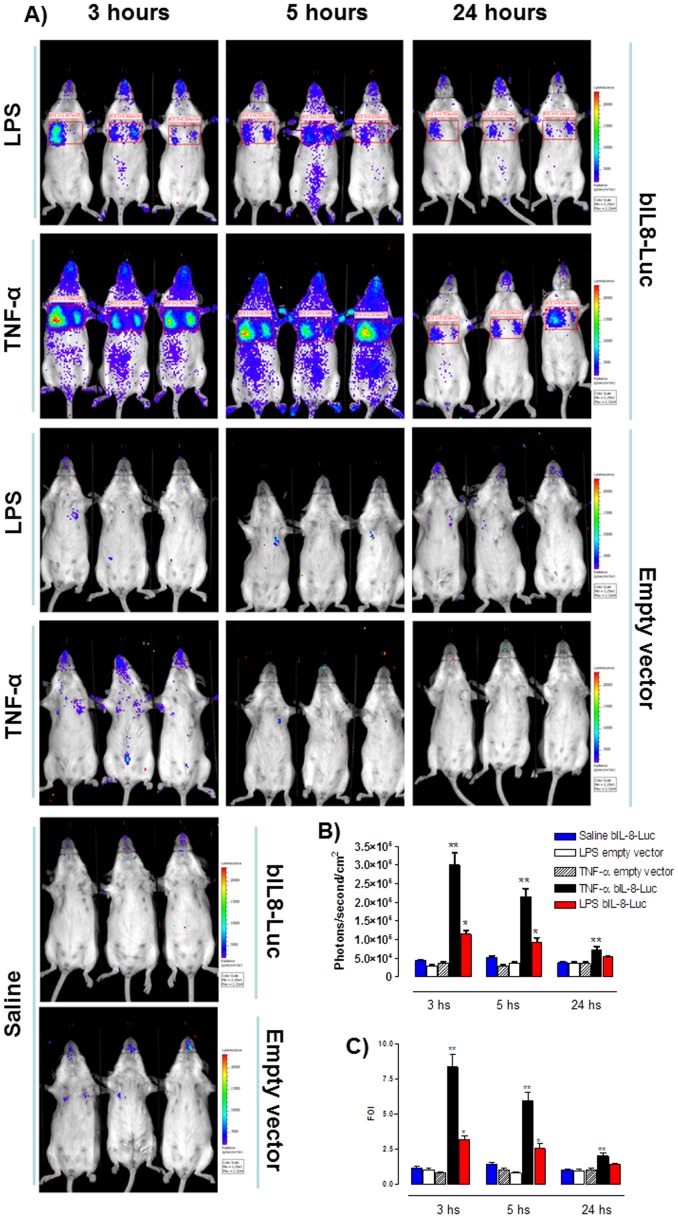
bIL-8-Luc reporter construct response *in vivo*. **A**) Representative images of groups of mice (n = 3 per group) transiently transgenized with bIL-8-Luc or Empty vector DNA and intratracheally instilled with LPS, TNF-α or vehicle (saline solution). Mice were monitored at 3, 5 and 24 hours post stimulation by BLI drawing a region of interest (ROI) over the chest. **B**) Light intensity quantification of the ROI using the LivingImage software. The experiment was repeated three times and each point represents the mean ± standard deviation of 9 animals. **C**) Data were expressed as folds of induction over vehicle-treated mice (Saline) and statistical differences were tested by One Way ANOVA followed by Dunnet’s post hoc test for group comparisons. Results are reported as mean ± SD and significance attributed when *P*<0.05 (*) or *P*<0.01(**).

### The Bovine IL-8 Promoter is Responsive in Mouse Lung *in vivo*

Given the positive results obtained *in vitro*, bIL-8-luc reporter construct was tested *in vivo*. Mice were first transiently transgenized with bIL-8-luc or the Empty vector control (pGL3 basic) and 10 days post transgenization were intratracheally instilled with LPS or TNF-α. At different time points (3, 5 and 24 hours) post instillation mice were monitored for luciferase expression in the lung by *in vivo* image analysis. Mice responded to both treatments with a robust induction already at 3 hours after instillation and the response still remained significant after 24 hours. In contrast no signal was observed for control mice treated with saline or transiently transgenized with the Empty vector (**Fig. 2A and B**). Signal intensity was then expressed as folds of induction where the untreated control was considered as 1 (**Fig. 2C**). A further control was established with mice transiently transgenized with different promoter reporter constructs such as STAT-1-Luc and ELK-1-Luc and no signal was detected after LPS or TNF-α treatment (**Figure S1**).

This *in vivo* analysis demonstrated the striking specificity of the system and a better induction with a lower background compared to the data obtained *in vitro*. Results are reported as mean ± SD and significance attributed when *P*<0.05 (*) or *P*<0.01(**).

### Long Lasting Response of Bovine IL-8 Reporter Gene *in vivo*

**Figure 3:**
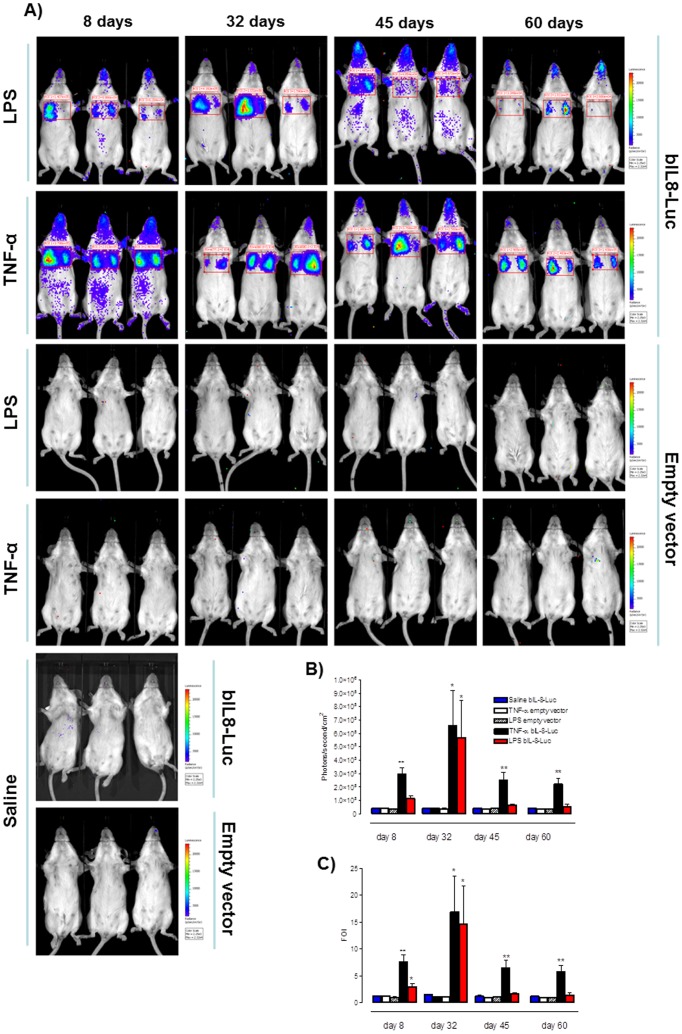
Long lasting response of bIL-8-Luc reporter construct. **A**) Representative images of groups of mice (n = 3 per group) transiently transgenized with bIL-8-Luc or Empty vector DNA and intratracheally instilled with LPS, TNF-α or vehicle (saline solution) at 8, 32, 45 and 60 days post transgenization. Mice were monitored at 3 hours post stimulation by BLI by drawing a region of interest (ROI) over the chest. **B**) Light intensity quantification of the ROI using the LivingImage software. The experiment was repeated three times and each point represents the mean ± standard deviation of 9 animals. **C**) Data were expressed as folds of induction over vehicle-treated mice (Saline) and statistical differences were tested by One Way ANOVA followed by Dunnet’s post hoc test for group comparisons. Results are reported as mean ± SD and significance attributed when *P*<0.05 (*) or *P*<0.01(**).

Similarly to *in vitro* transient transfections, the gene delivery system employed *in vivo* is expected to result in a transient expression of the delivered bIL-8-Luc reporter construct. It was therefore important to assess the extent and the duration *in vivo* of the responsiveness of the bIL-8-Luc reporter construct. Mice transiently transgenized with bIL-8-Luc or the Empty vector were intratracheally instilled with LPS or TNF-α at 8, 32, 45 and 60 days post transgenization and 3, 5, or 24 h post treatment were monitored for luciferase expression in the lung by *in vivo* image analysis. Surprisingly, transiently transgenized mice responded to LPS or TNF-α up to 60 days after transfection (**Fig. 3**) with the greater response at 32 days. In contrast, no signal was detectable in mice treated with saline or transiently transgenized with the Empty vector. Results are reported as mean ± SD and significance attributed when *P*<0.05 (*) or *P*<0.01(**).

Thus, our data suggest that the same mice can be utilized or reutilized for at least 45 days after the administration of the reporter gene, saving animals and costs. This will offer the opportunity of monitoring every single animal over an extended time period.

### Bovine IL-8 Reporter Gene Response Correlates with White Blood Cells and Neutrophils Infiltration

**Figure 4:**
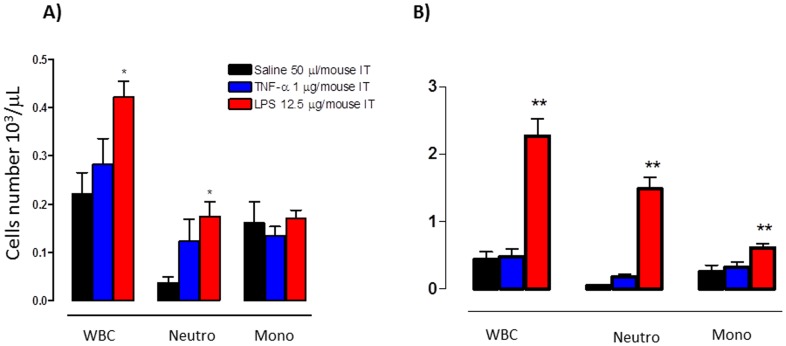
White blood cells and neutrophils infiltration. Cellular infiltration into the lung of mice intratracheally instilled with LPS, TNF-α or vehicle. The amount of White blood cells (WBC), Neutrophils (Neut) and Monocytes (Mono) found in BALF was expressed as number of cells per μl at 3 (A) and 24 hours (B) post treatment. The experiment was repeated three times and each point represents the mean ± standard deviation of 9 animals. Data were expressed as folds of induction over vehicle-treated mice (Saline) and statistical differences were tested by One Way ANOVA followed by Dunnet’s post hoc test for group comparisons. Results are reported as mean ± SD and significance attributed when *P*<0.05 (*) or *P*<0.01(**).

Since IL-8 is a major chemotactic polypeptide for neutrophils, we examined the correlation between bIL-8-Luc activation and neutrophils infiltration into the lung upon LPS or TNF-α stimulation. Ten days post bIL-8-Luc transient transgenization, the mice were intratracheally instilled with LPS, TNF-α or saline solution. Three hours post treatment, which was the time corresponding to the maximal bIL-8-luc activation and at 24 h post treatment, corresponding to the decline of the signal, the lungs of the treated mice were intratracheally washed and the broncho-alveolar lavage fluid (BALF) was assayed for the presence of infiltrating total white blood cells (WBC) and neutrophils. As shown in **Fig. 4**, WBC and neutrophils significantly increased in treated mice with respect to the untreated control, whereas the number of monocytes remained constant, a finding in line with the notion that monocytic infiltration is typically a late event of inflammatory responses. However, in terms of cellular infiltration response, LPS gave a better response than TNF-α. Thus a positive correlation between bIL-8-Luc reporter construct activation and white blood cells and neutrophils infiltration was observed. Results are reported as mean ± SD and significance attributed when *P*<0.05 (*) or *P*<0.01(**).

## Discussion

A hallmark of tissue inflammation following chemical, physical or microbiological insults is the recruitment of white blood cells to the injured tissue, which is orchestrated by chemotactic polypeptides named chemokines, among which IL-8 is one of the most remarkable. A significant correlation between IL-8 levels and neutrophil infiltration in diseases has been reported. IL-8 is an essential factor for acute inflammation and induces the infiltration of T lymphocytes into inflamed tissue. Recent studies have also demonstrated that IL-8 is involved in non-inflammatory reactions like angiogenesis [18]. In addition, systemic administration of IL-8 rapidly induces migration of hematopoietic stem cells from bone marrow to peripheral blood [19]. These results suggest that IL-8 also regulates non-inflammatory physiological reactions *in vivo*.

L-8 transcriptional activation is closely correlated with the initiation of acute inflammatory responses and thus is an ideal indirect read-out for monitoring the activation of inflammatory pathways targeting IL-8 expression. IL-8 homologs in many species have been cloned, including human [12], macaque [20], Sooty mangabey [21], bovine [22], sheep [23], pig [24], dog [25], rabbit [26], Guinea pig [27] and chicken [28]; however, a homolog of IL-8 has not been described in small rodents, including mice. In this study we developed a useful murine model for monitoring IL-8 expression in vivo. To this aim the bovine IL-8 promoter was chosen for two main reasons. First, lung inflammation-associated diseases in ruminants are very important in terms of economic loss for the beef meat industry; according to a report of the Committee for Animal Health Research Programs, major disease-related losses attributed to respiratory infections were calculated to be ∼400 million Euros per year; therefore the creation of an innovative in vivo platform for testing new respiratory anti-inflammatory drugs targeting specific pathways, such as IL-8, would be highly desirable. Second, all the information obtained on IL-8 bovinized mice can be easily employed for the human lung inflammation associated diseases, due to the high similarity existing between respiratory diseases in ruminants and human.

Although the minimal responsive sequence for the bovine IL-8 promoter is represented by the first 200 bp starting from the putative transcriptional initiation site, a 2 kb large version of the promoter was utilized [17] in order to include most of the potentially relevant binding sites for transcription factors. Moreover, recently two IL-8 promoter haplotypes have been characterized: the type 1, less sensitive to LPS and TNF-α and the type 2 with higher sensitivity to LPS and TNF-α [29].

Because IL-8 was previously shown to be expressed by a human epithelial cell line derived from lung after specific stimuli [30], [31], the bIL-8 reporter construct was initially tested *in vitro* in a mouse lung epithelial cell line, LA-4, which expresses TLRs and TNF-αRs. Although mice and rats do not have IL-8 gene, bovine IL-8 promoter/luciferase reporter construct was expressed at detectable levels in transfected LA-4 cells and was significantly induced upon treatment with LPS or TNF-α. This simple but extremely informative experiment demonstrates that mouse cells have the transcriptional machinery for transactivating a heterologous IL-8 promoter/luciferase reporter construct.

To functionally test the IL-8 promoter/luciferase reporter construct in mice, an *in vivo* bioluminescent imaging (BLI) approach was exploited. BLI is a sensitive tool that is based on the detection of light emission from cells or tissues [32]. The utility of reporter gene technology makes it possible to analyze specific cellular and biological processes in a living animal through *in vivo* imaging methods. Bioluminescence from luciferase gene expression, as is the case of the reporter construct employed during this study, is the most widely used. Because mammalian tissues do not naturally emit bioluminescence, *in vivo* BLI has considerable appeal because images can be generated with absence of background signal. BLI requires an expression cassette consisting of the bioluminescence reporter gene (in this case was luciferase) under the control of a selected promoter (in this experimental setting a bovine IL-8 gene promoter) driving the reporter. In order to induce light production, the substrate luciferin must be provided by intravascular or intraperitoneal injection. To date, there have been no reports of toxicity related to repeated dosing of substrates [32].

Although delivery of cationic polyethylenimine (PEI)/DNA complex could cause mortality when injected in mice in a strain dependent manner, we observed 100% survival rate when bIL-8-luc DNA was intravenously injected in FVB mice. Furthermore, a more robust fold-induction in response to LPS or TNF-α was obtained in the lung of bIL-8-luc DNA injected mice than in bIL-8-luc DNA transfected LA-4 cells. Indeed, bIL-8-luc DNA may have transfected several lung cell types, leading to the signal amplification orchestrated by the complex cellular network of the lung parenchyma. Although this is an important issue to be explored, it is out of the scope of the present study.

Noteworthy, bIL-8-luc transiently transgenized mice could be stimulated with LPS or TNF-α up to 60 days. Thus, the capability to monitor a biological process longitudinally in the same mouse represents an obvious advance for functional as well as pharmacological studies. The correlation between WBC and neutrophils infiltration and luciferase expression following LPS or TNF-α stimulation is in accordance with the recognized IL-8 role as a key factor in orchestrating inflammation, thus further validating the association between IL-8 transcriptional activation and pulmonary inflammation in the experimental setting that we here adopted. Although a bovine promoter was used in this study as a proof of concept, the results of the study suggest that IL-8 promoter regions derived from other species, including primates, could also be utilized in this experimental setting.

IL-8 exerts a wide variety of actions on pulmonary disease pathophysiology in ruminants and human during bacterial or viral infection, acute respiratory distress syndrome (ARDS), reperfusion injury and transplantation, lung injuries due to physical and chemical conditions, allergic inflammation and asthma, idiopathic pulmonary fibrosis and other diffuse lung diseases, including lung cancer [2], [4]. The absence of IL-8 and its receptor orthologs in small rodents hindered the possibility of testing novel strategies targeting IL-8 using mice as a convenient small size experimental animal model. We filled the gap by generating a mouse model transiently expressing a reporter gene under the control of an IL-8 promoter which is suitable to functionally study IL-8 promoter in a convenient small size experimental animal model.

## Materials and Methods

### Construct

bIL-8-Luc was obtained by sub-cloning the 2030 bp IL8 bovine promoter, amplified by PCR from Madin-Darby bovine kidney (MDBK; ATCC #CCL-22) genomic DNA and subcloned into the digested pGL3basic vector (Promega) as previously described [17]. Whereas STAT-1-Luc and ELK-1-Luc containing the minimal promoter of STAT-1 and ELK-1 human gene were obtained from Switchgear Genomics.

### Cell Cultures

Mouse lung adenocarcinoma cell line of epithelial origin, LA-4 cells (ATTC, #CCL-196), was cultured in Dulbecco's modified essential medium (Sigma) containing 10% FBS, 2 mM of L-glutamine, 100 IU/ml of penicillin, 100 µg/ml of streptomycin, and 2.5 µg/ml of amphotericin B (Sigma). Cell culture plates or flasks were incubated at 37°C with 5% CO_2_ in air, in a humidified atmosphere.

### LA-4 Cells Characterization by RT-PCR for TNF-αRI and II and TLRs

Total RNA from LA-4 cells was extracted with TriPure reagent (Roche) and 5 μg of total RNA were reverse transcribed using Ready-To-Go, T-Primed First-Strand Kit (Amersham Biosciences). Two microliters of the resulting cDNA were amplified in a final volume of 50 μl of 10 mM Tris–hydrochloride, pH 8.3, containing 0.2 mM deoxynucleotide triphosphates, 2.5 mM MgCl_2_, 50 mM KCl, 1 U of Taq polymerase (Invitrogen) and 0.25 μM of each primer listed in **Table S1**. The amplification program for TNF-αRI, II and GAPDH was 35 cycles, each cycle consisting of denaturation at 94°C for 1 min, primer annealing at 55°C for 1 min, and chain elongation at 72°C for 1 min [33]. The amplification program for TLRs was 35 cycles, each cycle consisting of denaturation at 94°C for 1 min, primer annealing at 55°C for 1 min, and chain elongation at 72°C for 1 min [34]. PCR products were analyzed on a 2% agarose gel.

### LA-4 Cell Electroporation

LA-4 cells were cultured in a 75 cm^2^ flask. When growth reached 90% confluence cells were electroporated. bIL-8-Luc plasmid DNA (20 µg) diluted in 600 µl of DMEM without serum was electroporated (Equibio apparatus Wolf Laboratories Limit., York, UK) into LA-4 cells at 186 V, 960 µF, in 4-mm gap cuvettes (Wolf Laboratories Limit., York, UK)). Electroporated cells were plated in a twenty four-well plates and incubated in an atmosphere of 95% air and 5% CO_2_ at 37°C.

### Dual Luciferase Reporter Assay

Twenty-four hours post electroporation, LA-4 cells in twenty four-well plates were treated with LPS (500 ng/ml) (Sigma) or TNF-α (10 ng/ml) (Sigma) for four hours.

The luciferase reporter assay was performed with a Dual Luciferase Reporter Assay System kit (Promega Corp., Madison, WI, USA) according to the manufacturer’s specification with minor modifications. Following treatment, cells were washed with PBS and lysed with 100 µl of passive lysis buffer by freeze-thawing at −80°C. Ten microliters of the cell lysate were added to 50 µl of LAR and Luciferase activity was determined with a PerkinElmer Victor^3^ Multilabel Counter (PerkinElmer, Waltham, MA, USA), according to the manufacturer’s specifications. Experiments were performed with 4 replicates at each time point and each experiment repeated three times. Statistical differences were tested by One Way ANOVA followed by Dunnet’s post hoc test for group comparisons.

### Experimental Animals

Female inbred FVB (7–8 week-old) mice were purchased from Harlan Laboratories Italy (San Pietro al Natisone, Udine). Animals were maintained under conventional housing conditions. Prior to use, animals were acclimatized for at least 5 days to the local vivarium conditions (room temperature: 20–24°C; relative humidity: 40–70%), having free access to standard rat chow and tap water. All experiments were carried out in rodents and exclusively included painless suppression of animals. The experiments comply with the Principles of Animal Care (publication no. 85–23, revised 1985) of the National Institutes of Health and with the current law of the European Union and Italy (D. L.vo 116/92). The present project was approved by the Ethical Committee of the University of Parma (Italy).

### *In vivo* Gene Delivery

We applied *in vivo* JetPEI (Polyplus Transfection) as a carrier for delivering DNA to lung tissues. The DNA and JetPEI mix was formulated according to the product manual with a final N/P ratio of 7. Briefly, 40 μg of bIL-8–luc, STAT-1-Luc, ELK-1-Luc or pGL3 basic reporters and 7 μL of JetPEI were each diluted into 200 μL 5% glucose. The two solutions were then mixed and incubated for 15 minutes at room temperature. The entire mixture was i.v. injected into FVB mice and the expression of bIL-8–Luc, STAT-1-Luc, ELK-1-Luc or pGL3 basic reporters was monitored through imaging with IVIS.

### *In vivo* Bioluminescence Imaging (BLI)

*In vivo* imaging was performed using an IVIS imaging system (Caliper Life Sciences, Alameda, CA). From 10 days after DNA delivery the transient transgenic mice were imaged in order to check the baseline activation of the IL-8 pathway. The day after transgenization, the mice were intratracheally challenged with LPS (12.5 µg/mouse) or TNF-α (1 µg/mouse) and the lungs imaged using bioluminescence (BLI) after 2, 4, 7 and 24 hours following intraperitoneal injection of 150 mg/kg luciferin. The mice were anesthetized with gas anaesthesia (isoflurane 2.5%), imaged for 5 minutes, 10 minutes and 15 minutes after luciferin injection in order to minimized the pharmacokinetic variability among mice. Photons emitted from specific regions were quantified using Living Image® software (Caliper Life Sciences, Alameda, CA).

### Bronchoalveolar Lavage Fluid (BALF) and Cell Counting

Bronchoalveolar lavage (BAL) was performed as previously described [35]. Briefly, mouse tracheas were cannulated with an 18-gauge angiocathether (Becton Dickinson). Six hundred microliters of sterile HANK’S buffer were instilled three times in the bronchial tree and collected for subsequent analysis. After centrifugation at 400 g for 10 minutes, the cell pellet was resuspended in 0.2 ml of PBS. Total cell number was counted with a particle counter (Dasit XT 1800J). The pellets deriving from the same animal were combined and resuspended in a volume of 0.2 ml and total and differential cell counts were performed within 2 hours using an automated cell counter (Dasit xt 1800 J, Sysmex). The cell count per animal was calculated from the number of cells for 1 µl of BALF multiplied for the volume used for the re-suspension of the cell pellet.

### Statistics

Experiments were performed with 4 replicates at each time point and each experiment repeated three times. Data were analysed using One Way ANOVA followed by Dunnet’s post hoc test for group comparisons. Results are reported as mean ± SD and significance attributed when *P*<0.05 (*) or *P*<0.01(**).

## Supporting Information

Figure S1 **Representative images of groups of mice (n = 3 per group) transiently transgenized with ELK-1-Luc or STAT-1-Luc DNA and intratracheally instilled with LPS or TNF-α.** Mice were monitored at 3, 5 and 24 hours post stimulation by BLI and no signal was detectable.
(TIF)

Table S1 List of primers used in this work.
(DOC)
