# Supplementary figures and images for: In Vivo Imaging of Transiently Transgenized Mice with a Bovine Interleukin 8 (CXCL8) Promoter/Luciferase Reporter Construct
Source: PLoS One. 2012 Jun 28;7(6):e39716. doi: 10.1371/journal.pone.0039716 (PMC3386280; doi:10.1371/journal.pone.0039716)

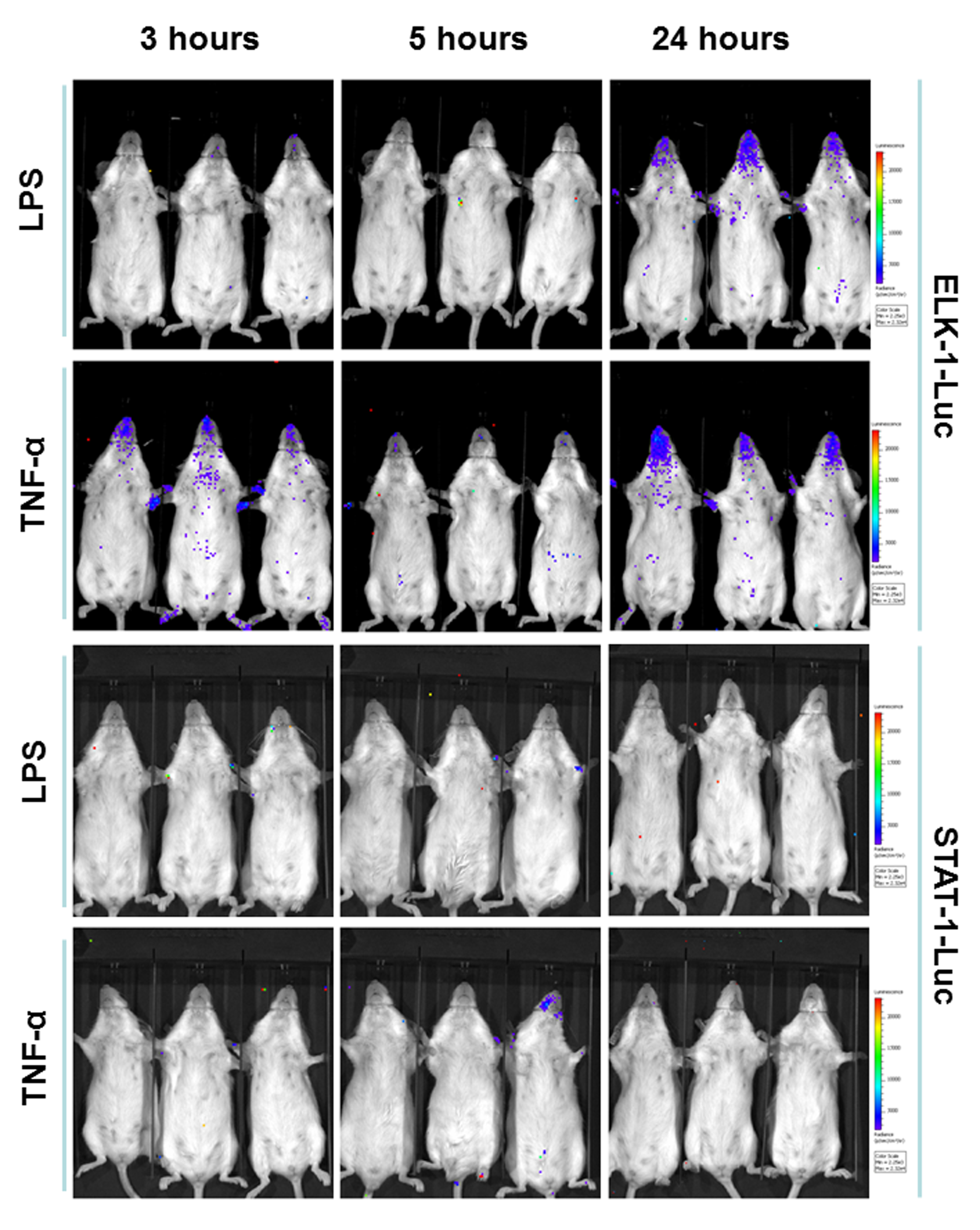

Supplement: Figure S1 — Representative images of groups of mice (n = 3 per group) transiently transgenized with ELK-1-Luc or STAT-1-Luc DNA and intratracheally instilled with LPS or TNF-α. Mice were monitored at 3, 5 and 24 hours post stimulation by BLI and no signal was detectable. (TIF) [file pone.0039716.s001.tif]
