# Supplementary material for: In Vivo Imaging of Transiently Transgenized Mice with a Bovine Interleukin 8 (CXCL8) Promoter/Luciferase Reporter Construct
Source: PLoS One. 2012 Jun 28;7(6):e39716. doi: 10.1371/journal.pone.0039716 (PMC3386280; doi:10.1371/journal.pone.0039716)
Supplement: Table S1 — List of primers used in this work. (DOC) [file pone.0039716.s002.doc]

| **Primer name** | **Primer sequence** | **amplicon** |
| --- | --- | --- |
| mTLR1-sense | 5’-caa cag tca gcc tca agc att-3’ | 190bp |
| mTLR1-anti | 5’-taa gca tct cct aac acc agc a-3’ |
| mTLR2-sense | 5’-ctg gag aag gtg aag cga at-3’ | 432bp |
| mTLR2-anti | 5’-ccg agg caa gaa caa aga aa-3’ |
| mTLR3-sense | 5’-att ctc cct tgc tca ctc tca-3’ | 346bp |
| mTLR3-anti | 5’-atg ttg gct atg ttg ttg ttg c-3’ |
| mTLR4-sense | 5’-gct ttc acc tct gcc ttc act a-3’ | 395bp |
| mTLR4-anti | 5’-cgt ttc ttg ttc ttc ctc tgc t-3 |
| mTLR5-sense | 5’-gac tgc gat gaa gag gaa gc-3’ | 494bp |
| mTLR5-anti | 5’-acc acca cc acg atg aga at-3’ |
| mTLR6-sense | 5’-ata ctc ggt gtt tgc tga gat g-3’ | 410bp |
| mTLR6-anti | 5’-tgt tgt tgt gaa ggt caa gga c-3’ |
| mTLR7-sense | 5’-cct tct ttc ttg cct ttg aat g-3’ | 450bp |
| mTLR7-anti | 5’-taa atg gtt gcc tct gaa ctc c-3’ |
| mTLR8-sense | 5’-tat ttg ggc tgg aac tgc tat t-3’ | 313bp |
| mTLR8-anti | 5’-gag gat gta tgt gga tgg atg a-3’ |
| mTLR9-sense | 5’-gaa agc atc aac cac acc aat-3’ | 304bp |
| mTLR9-anti | 5’-aca agt cca caa agc gaa gg-3’ |
| mGAPDH-sense | 5’-cag gtt gtc tcc tgc gac tt-3’ | 212bp |
| mGAPDH-anti | 5’-ctt gct cag tgt cct tgc tg-3’ |
| mTNFRI-sense | 5’-gtg cct acc tcc tcc gct t-3’ | 503bp |
| mTNFRI-anti | 5’-cag cat aca gaa tcg caa ggt c-3’ |
| mTNFRII-sense | 5’-gca aca aga cct cgg aca c-3’ | 539bp |
| mTNFRII-anti | 5’-tga acc caa cga tgt aag ga-3’ |
